# A Positive Sign of Pregnancy during the First Three Months

**Published:** 1880-11

**Authors:** J. H. Carstens

**Affiliations:** Lecturer on Clinical Medicine, etc., Detroit Medical College


### A POSITIVE SIGN OF PREGNANCY DURING THE FIRST THREE MONTHS.

By J. H. CARSTENS, M.D.,
Lecturer on Clinical Medicine, etc., Detroit Medical College.

The difficulty of diagnosing pregnancy during the earlier months is well known, and a positive and unfailing sign would be of great value. Reading in a late number of the *American Journal of Obstetrics* of a discussion, which took place in the Boston Obstet. Society on this subject, and finding no mention made there, nor in the text books in general use, of a positive sign on which I have always relied, and which has in my experience never failed to enable me to make a diagnosis, it occurred to me to call your attention to this question. I was under the impression that it was a new, and heretofore not described sign, but looking over the literature of obstetrics, I found that it has been mentioned years ago by Jacquemier and Kluege, but it seems to have fallen into oblivion, and is not mentioned in the ordinary text books.

I refer to the color of the mucous membrane of the vagina and cervix uteri. This I have always found of a purplish blue, or rather deep violet hue, in pregnant women, and I have depended on this peculiar color in making a diagnosis of pregnancy in the first, second and third month. I say it has never failed and it is not produced by any pathological condition, the different colors produced by uterine diseases cannot be mistaken for this pathognomonic violet hue. I have often called the attention of students to this sign, and in dispensary practice it has repeatedly occurred that women under my treatment for uterine diseases have not attended for six or eight weeks, and hastily placing them on a table without inquiring about their last menstruation, I introduced a speculum, and was on the point of introducing a probe, or making an application to the uterus, when behold, there was the characteristic color. I desisted from further interference, and in every case which I could keep under observation the women were afterwards delivered in full term, or had a miscarriage.

I have also been prompted to write this paper on account of a case lately under my observation, which puzzled me, and the other physician called, the details of which I shall write up some other time.

The case was very peculiar, a woman under my treatment for endometritis and subinvolution. During the course of the treatment menstruation ceased, she claimed she was pregnant, but as I had applied various remedies to the mucous membrane up to the very fundus of the uterus, and continued to do so for some months, I insisted that she was not pregnant, and that it was impossible for her to be so. This continued for about five months, she claiming one thing and I denying it. Well, this woman had the peculiar violet discoloration, and I often asked myself the question, "Here is a case with the peculiar, and in your opinion, pathognomonic sign of pregnancy, and you say she is not in a family-way, how is this?" The vision of some day writing an article of value for the *American Journal of Obstetrics* suddenly vanished.

"Here," I said to myself, "is a case with the deep violet hue of the mucous membrane, she has other signs of pregnancy, but she is not pregnant, for you pass your probe readily to the fundus, your sign is not infallible." But it occurred to me that it might be a case of tubal or extra-uterine pregnancy, and I watched the case with great interest. One day I was called in haste, and imagine my feelings, when, arriving at the bedside, I found between the thighs of the woman a five months dead fœtus with the placenta still inside of the uterus. However unsatisfactory the case was otherwise, it, however, has strengthened my now unfailing faith in the sure sign of pregnancy, the violet hue of the mucous membrane of the genital organs.

It has been claimed by some that this color of the mucous membrane has been found in various pathological states. I claim that the discoloration in the latter case is dfferent from that found during pregnancy, it is more blue and scarlet, mixed or dotted, nor is the peculiar soft, velvety condition of the membrane present. I can simply call it violet, it must be seen and then never will be forgotten. It is probably caused by engorgement of the veins.

All I ask is that this sign be again looked to and submitted to a rigid investigation, and I am sure the verdict will be that it is the only sure sign we have at present to diagnose pregnancy from the first few weeks up to the fourth month. It has never failed me, I have often staked my reputation on it, but when I failed to heed the warning color I came to grief.—*Detroit Lancet.*

